# A Bimaterial Beam Strategy for Suppressing Thermal Deformation of Arc-Shaped CFRP Ribs via Asymmetric Laminate Design

**DOI:** 10.3390/ma19143137

**Published:** 2026-07-22

**Authors:** Yonggang Xue, Xiaofei Ma, Yonggang Fang, Dayu Zhang, Jialong Zhu, Pengbo Su

**Affiliations:** 1 College of Astronautics, Nanjing University of Aeronautics and Astronautics, Nanjing 210016, China; xiaoyg_2025@163.com; 2 Xi’an Institute of Space Radio Technology, Xi’an 710100, China; saintfyg@163.com (Y.F.); zhangdayu504@foxmail.com (D.Z.); 18092166275@163.com (J.Z.); su_pengbo@126.com (P.S.)

**Keywords:** asymmetric laminate, thermal deformation, CFRP, CTE, bimaterial beam, common-mode rejection, space structures

## Abstract

Deployable reflector antennas demand high geometric precision; the Ruze equation directly links surface error to RF gain. Arc-shaped CFRP ribs are vulnerable to thermal deformation, as their curvature converts in-plane expansion into out-of-plane displacement, which symmetric laminates cannot suppress. Classical laminate theory (CLT) underestimates the coefficient of thermal expansion (CTE) of cross-ply laminates by factors of 1.75–2.38 for the laminate configurations investigated in this study, causing up to 79.4% of displacement prediction errors in symmetric designs. Here, we present an asymmetric laminate that overcomes both limitations. The upper skin (nine plies) and lower/web skins (seven plies) from the same prepreg batch create a CTE mismatch (Δα = 6.30 × 10^−7^ K^−1^), activating coupling stiffness to generate a thermal moment opposing curvature-driven displacement. Because both skins share identical batch history, CTE prediction errors cancel through common-mode rejection. Compared with the symmetric design, the asymmetric design achieved a 50.4% reduction in thermal deformation (from 210 µm to 104 µm) and improved FEA accuracy from 79.4% error to 4.8% error under experimental schemes. The method uses only conventional 0/90° prepreg and standard autoclave processing, with the upper-surface ply count as the sole design variable for a given section’s geometry, establishing retained coupling stiffness as a practical route to dimensional stability in curved space structures.

## 1. Introduction

Deployable reflector antennas are critical payloads for communication and Earth-observation satellites; their electrical performance depends on the geometric accuracy of the reflecting surface—the Ruze equation relates gain loss to the root-mean-square surface deviation normalized to the wavelength [1,2,3]. In low Earth orbit, antenna structures experience a temperature swing of 160 °C, ranging from approximately −100 °C during eclipse to +60 °C under direct solar exposure. The arc-shaped ribs that form the primary load-bearing framework are particularly susceptible to thermal deformation because their curvature converts in-plane thermal expansion into out-of-plane normal displacement, a kinematic effect that cannot be suppressed by laminate symmetry alone [4]. Classical laminate theory (CLT) relates ply-level properties to laminate behavior through the [A], [B], and [D] matrices [5,6,7,8,9]. In engineering practice, dimensional stability is typically achieved through mid-plane symmetric layup ([B] = 0), which decouples in-plane expansion from bending [5,6,8]. For flat panels, this is effective; however, for arc-shaped ribs, the structural curvature distorts the displacement field, and symmetry alone provides no mechanism to counteract the curvature-driven normal displacement [4].

A further limitation of the symmetric design is that CLT systematically underestimates the effective laminate CTE for the cross-ply configurations considered in this work. The theory assumes perfect inter-ply bonding and does not account for three categories of manufacturing effects: fiber misalignment during hand layup, resin-rich inter-ply layers, and residual stresses locked in during autoclave curing [10,11]. In this work, the CLT-predicted chord-direction CTE of the 7-ply [0/90] laminate was 8.15 × 10^−7^ K^−1^, whereas the measured value reached 1.943 × 10^−6^ K^−1^, a factor of 2.38. Because the symmetric design is directly CTE-driven, this discrepancy propagates into the displacement prediction error.

Alternative thermal deformation control strategies have been documented in the literature: variable-angle tow placement [12], near-zero CTE laminate designs [13,14,15,16], process-induced deformation compensation [17,18,19], and active thermal control [20,21]. These approaches address fiber path optimization, material hybridization, manufacturing adjustment, and post-manufacture correction, but the central difficulty remains that CLT does not accurately predict the effective CTE of multi-ply laminates, and this error propagates into the FEA displacement prediction. The objective of the present work was to address this difficulty not by seeking a more accurate CLT prediction, but by designing a laminate architecture in which the displacement is governed by a parameter that is intrinsically more stable than the absolute CTE; that is, the CTE mismatch between two laminate skins that share the same manufacturing history.

## 2. Materials and Methods

### 2.1. Material and Laminate CTE Measurement

**Figure 1:**
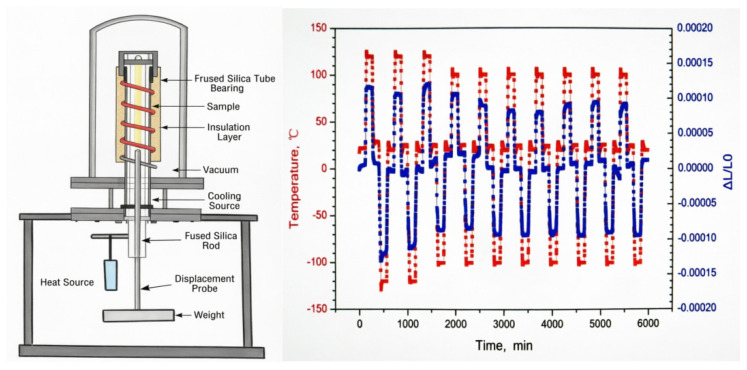
Schematic of the CTE measurement setup and corresponding test curves.

**Figure 2:**
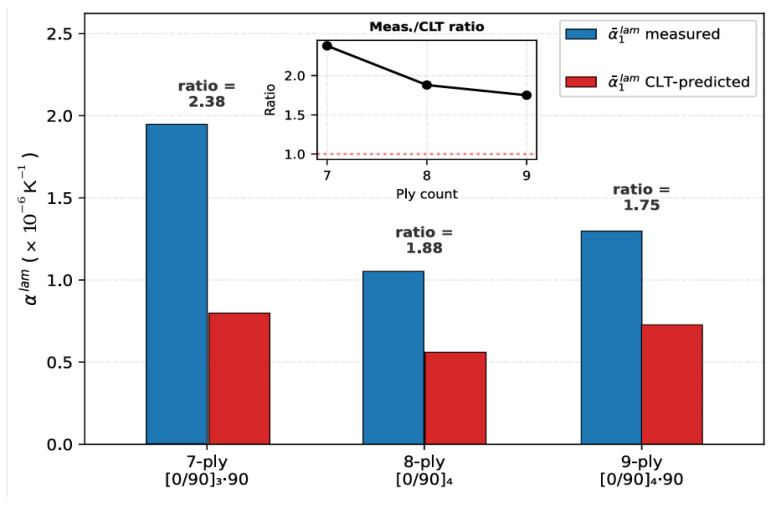
Measured vs. CLT-predicted laminate CTE. The dashed line is the baseline (ratio = 1) for comparison.

M40J carbon fiber/BA9913 epoxy unidirectional prepreg was used throughout. The nominal ply thickness was 0.11 mm. Measured UD-ply properties are listed in Table 1. Three multidirectional [0/90] laminates (7-ply, 8-ply, and 9-ply) were fabricated and tested on 200 mm (L) × 25 mm (W) × 3 mm (H) specimens using a custom-built dilatometer. The dilatometer employed a linear variable differential transformer (LVDT) with a resolution of 0.1 μm, and was calibrated against a fused silica reference standard (NIST-traceable) over the temperature range of −150 °C to +150 °C (Figure 1). The measurement uncertainty, evaluated from five repeat measurements on the fused silica reference standard under a high-vacuum environment (<1.33 × 10^−3^ Pa), was ± 0.2 μm/m/°C (k = 2, 95% confidence interval, Mechanics Laboratory, Harbin Institute of Technology, Harbin, China). Table 2 compares the measured laminate CTE values with CLT predictions. The measured-to-CLT CTE ratio was 2.38 for the 7-ply laminate, 1.88 for the 8-ply, and 1.75 for the 9-ply (Figure 2) [22].

### 2.2. Bimaterial Beam Model: Thermal Moment from CTE Mismatch

**Figure 3:**
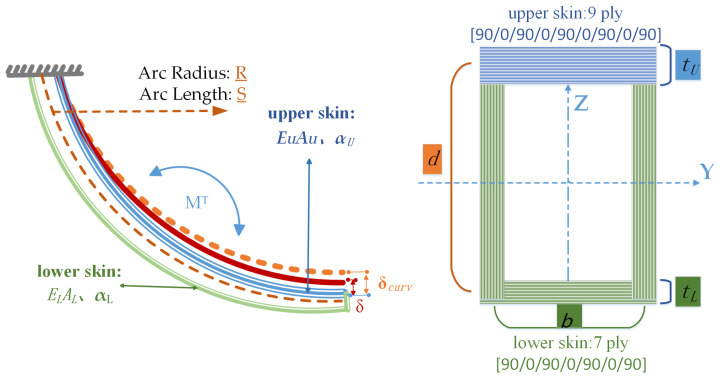
Bimaterial beam model schematic.

The rib cross-section is idealized as a box beam with two independently defined laminate skins. The upper skin occupies y ∈ (−b/2, b/2) and z ∈ (d/2 − t_U_, d/2 + t_U_), while the lower skin occupies y ∈ (−b/2, b/2) and z ∈ (−d/2 − t_L_, −d/2 + t_L_). Each skin is treated as a homogeneous plate with effective in-plane stiffness EA and CTE α. This simplification is standard in bimaterial beam analysis [23], and is valid when skin thickness is small relative to d (t_U_ ≈ t_L_ ≈ 0.77~0.99 mm and d = 42~80 mm). Figure 3 illustrates the bimaterial beam model.

The bimaterial beam effect is central to the asymmetric design [23,24]. When two laminate skins with different CTE values are bonded together, a temperature change ΔT generates a thermal bending moment *M^T^* that, for the box beam section, can be expressed as:

(1) $$M^{T}=\int_{A}E(z)\cdot \alpha (z)\cdot \Delta T\cdot Z\cdot dA\;$$

where M^T^ is the thermal bending moment (N·m); E is the elastic modulus at position z (Pa); and α denotes the coefficient of thermal expansion at position z (K^−1^).

Evaluating the integral separately for the upper and lower skins gives:

(2) $$M^{T}=E_{U}\cdot b\cdot t_{U}\cdot \alpha_{U}\cdot \Delta T\cdot \frac{d}{2}+E_{L}\cdot b\cdot t_{L}\cdot \alpha_{L}\cdot \Delta T\cdot \left(-\frac{d}{2}\right)$$

where E_U_ denotes the elastic modulus of the upper skin (Pa); E_L_ denotes the elastic modulus of the lower skin (Pa); α_U_ denotes the CTE of the upper skin (K^−1^); α_L_ denotes the CTE of the lower skin (K^−1^); and d denotes the distance between the upper and lower skin mid-planes (m).

Based on the measured chord-direction moduli (Table 2), the ratio of the two laminate skins in-plane tensile stiffness is E_U_A_U_/E_L_A_L_ = 1.34. The thermal bending moment for a box beam with upper and lower skins of different stiffness is:

(3) $$M^{T}=E\cdot A\cdot \Delta T\cdot \left[1.34\cdot \alpha_{U}\cdot \frac{d}{2}+\alpha_{L}\cdot \left(-\frac{d}{2}\right)\right]=E\cdot A\cdot \Delta T\cdot \frac{d}{2}\cdot \left(1.34\cdot \alpha_{U}-\alpha_{L}\right)$$

By noting that ∆α = α_L_ − 1.34α_U_, we obtain:

(4) $$M^{T}=-E\cdot A\cdot \Delta \alpha \cdot \Delta T\cdot \frac{d}{2}$$

where EA = E_L_A_L_ denotes the in-plane tensile stiffness of the lower skin (N); and Δα denotes the CTE mismatch between lower and upper skins (K^−1^).

Since Δα > 0 for the optimal configuration (the lower skin has a higher CTE), the negative sign in Equation (4) indicates that *M^T^* acts in the direction opposing the downward curvature-driven displacement. Equation (4) is the fundamental equation of the asymmetric design. For the symmetric baseline configuration where both skins have 7 plies, Δα = 0 and the thermal moment vanishes, leaving curvature-driven expansion as the only deformation mode. For the asymmetric layup where Δα ≠ 0, the thermal moment bends the rib upward if α_L_ > α_U_, opposing the downward curvature-driven displacement. The magnitude of the compensating moment is proportional to Δα, a larger section height or a larger CTE mismatch produces a stronger counterbalancing moment.

### 2.3. Tip Displacement Decomposition

The arc-shaped rib of radius R and arc length S undergoes two simultaneous deformations under thermal loading [25,26]. The total normal direction tip displacement is:

(5) $$\delta =\delta_{curv}+\delta_{therm}$$

where δ denotes the total normal direction tip displacement (m); δ_curv_ denotes the curvature-driven displacement component (m); and δ_therm_ denotes the thermal-moment-driven displacement component (m).

(6) $$\delta =\frac{S^{2}}{2R}\left(\alpha_{mean}\Delta T\right)+\frac{R^{2}\;\cdot \;\left(1-cos\left(S/R\right)\right)}{2EI}M^{T}$$

where S denotes the arc length of the rib (m); R denotes the arc radius of the rib (m); EI denotes the overall bending stiffness of the box section (N·m^2^); and α_mean_ = (α_L_ + α_U_)/2 = 1.628 × 10^−6^ K^−1^ is the arithmetic mean of the two skins’ CTE values.

Substituting Equation (4) into Equation (6) yields:

(7) $$\delta =\left[\frac{S^{2}}{2}\frac{\alpha_{mean}}{R}-\frac{R^{2}\;\cdot \;\left(1-cos\left(S/R\right)\right)\cdot EA\cdot \Delta \alpha \cdot }{2EI}\frac{d}{2}\right]\Delta T$$

The net displacement is the difference between two larger quantities of opposite sign. The bimaterial beam effect therefore acts as a displacement compensator, as the thermal moment does not eliminate the curvature-driven displacement but partially cancels it. For a given section’s geometry (fixed EI and d), the degree of cancelation is primarily controlled by a single design parameter, the upper-surface ply count, which determines Δα via the thickness-dependent CTE.

### 2.4. Finite Element Analysis and Experimental Validation

**Figure 4:**
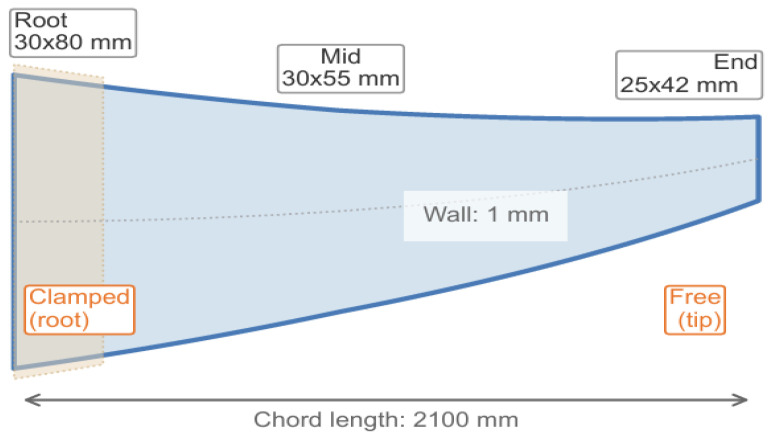
Rib geometry and layup design.

The arc-shaped CFRP antenna rib is modeled as a variable-cross-section thin-walled box beam with a chord length of 2100 mm; the typical cross-sectional dimensions at three locations are illustrated in Figure 4. Four surfaces (upper skin, lower skin, and two webs) were assigned independent [0/90] cross-ply layups, where the 0° direction aligns with the rib chord. Three layup schemes were designed to investigate the effect of ply-count asymmetry on thermal deformation [27]:

(i) Baseline (symmetric): All surfaces are 7-ply [0/90]_3_/0, with a total thickness 0.77 mm per wall;
(ii) Scheme 2: The upper skin is 8-ply [0/90]_4_, while the lower skin and webs are 7-ply, creating a negative CTE mismatch;
(iii) Scheme 3 (optimal): The upper skin is 9-ply [0/90]_4_/0, while the lower skin and webs are 7-ply, creating a positive CTE mismatch.

All specimens were fabricated from a single batch of M40J/BA9913 unidirectional prepreg, using hand layup followed by autoclave curing at 180 °C and 0.6 MPa for 2 h. The ply-count asymmetry was achieved by adding extra 0/90 plies to the upper surface only, keeping the lower skin and webs at the baseline 7-ply configuration. A screening experiment (n = 3 per configuration) was first conducted to compare the three layup schemes. Scheme 3 was selected as the optimal configuration based on the screening results, using the criterion of minimum tip displacement measured at +60 °C.

Finite element models of the three layup schemes were constructed using shell elements (15,204 nodes, 14,779 elements). A uniform temperature increase of +60 °C was applied. This value corresponds to the upper bound of the in-orbit temperature range and was chosen because it produces the largest thermal deformation in the positive-temperature regime, thereby providing the most stringent test of the compensation mechanism. The full orbital temperature swing of approximately 160 °C (from −100 °C to +60 °C) was cited in the Introduction to describe the operational environment; the +60 °C load case was selected for the FE analysis as a representative hot-case condition.

**Figure 5:**
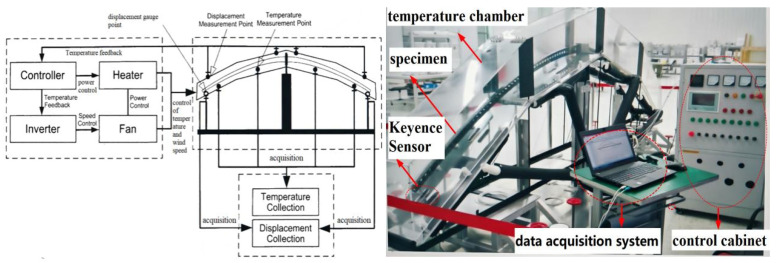
Eighteen full-scale rib specimens during the thermal deformation experiment.

A total of 18 full-scale rib specimens were manufactured from a single prepreg batch: 9 specimens for the screening phase (3 per layup configuration) and 9 specimens of the optimal Scheme 3 for validation (Figure 5). Thermal cycling was performed in a temperature chamber equipped with an Invar 4J36 fixture [28]. Displacement was measured using Keyence GT2-P12KL sensors (±1 µm accuracy, Keyence Corporation, Osaka, Japan). Each specimen underwent three thermal cycles; data from the second cycle (after internal stress relaxation) are reported.

## 3. Results

Table 3 presents the FEA-predicted displacements at +60 °C for the three layup schemes, together with the experimental means from the screening phase (n = 3 per configuration). The seven-ply symmetric baseline showed a mean displacement of 209.7 μm, whereas CLT-based FEA predicted only 43.3 μm [29], a 4.8-fold underestimation (79.4% error). Scheme 2 (8-ply upper) amplified the FEA displacement to 324 μm owing to its negative CTE mismatch (Δα < 0), which produced a thermal moment that reinforced the curvature-driven deformation; the experimental mean was 167.1 μm, yielding a 93.9% FEA error (overestimation). It is important to note that the large discrepancy for Scheme 2 is itself consistent with the CTE underestimation; because the FEA overestimates the magnitude of the CTE mismatch effect (using CLT-predicted CTE values that are systematically lower than the measured values), the predicted reinforcing moment is artificially large. The experimental results, being lower than the symmetric baseline, suggest that the actual CTE mismatch for the 8-ply/7-ply combination may be smaller than the CLT prediction, or that additional stiffness effects partially offset the reinforcing moment. This behavior warrants further investigation but does not undermine the validity of the bimaterial beam concept, which is confirmed by Scheme 3’s results.

Scheme 3 (9-ply upper) reduced the FEA-predicted displacement to 109 μm through a positive CTE mismatch (Δα > 0) that opposed the curvature effect, and the experimental mean of 131.0 μm gave an FEA error of 16.8%, the lowest among the three configurations. The measured-to-predicted CTE ratio of 2.38 for the 7-ply laminate decreased to 1.75 for 9-ply one (Figure 2).

This trend may be attributable to the presence of resin-rich inter-ply layers of approximately constant absolute thickness, whose relative contribution diminishes as the total laminate thickness increases. However, this explanation remains a hypothesis at present, as direct microscopic evidence of the inter-ply layer thicknesses has not been obtained. The 2.38-fold CTE underestimation of the 7-ply laminate contributes to the 4.8-fold FEA underestimation of the symmetric baseline displacement. Based on these screening results, Scheme 3 was selected for validation.

Table 4 lists the individual displacement measurements for the nine Scheme 3 validation specimens. The mean displacement was 104 μm with a standard deviation of 33.4 μm (range: 61 to 165 μm). The considerable scatter (coefficient of variation = 32%) likely reflects specimen-to-specimen variations in prepreg batch properties, hand-layup-induced fiber misalignment, and local thickness variations. Table 5 compares the FEA prediction of 109 μm with the experimental mean, while the absolute deviation was 5 μm, corresponding to a relative deviation of 4.8%.

**Figure 6:**
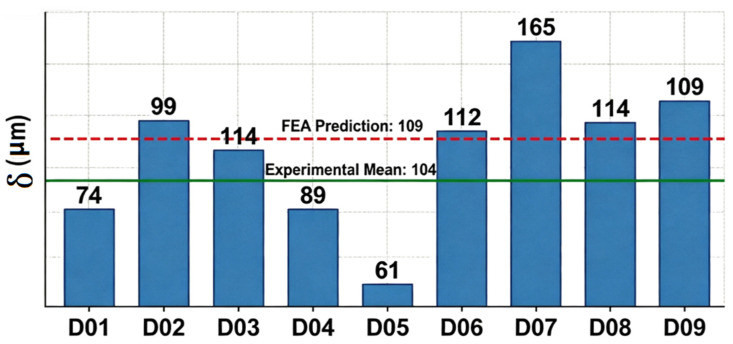
FEA prediction vs. experimental mean (4.8% deviation).

The 104 μm mean displacement of Scheme 3’s validation set confirms the bimaterial beam design principle. Relative to the symmetric baseline (209.7 μm from screening), the asymmetric design reduced thermal deformation by 50.4% in the validation phase [30]. The FEA prediction accuracy improved from 79.4% error (symmetric baseline, screening) to 4.8% error (Figure 6). This improvement is consistent with the common-mode rejection mechanism: the CTE mismatch Δα between same-batch skins is governed by the ratio of the CTE error factors rather than their absolute values. Specifically, if CLT overestimates (or underestimates) the CTE of both skins by similar multiplicative factors k_U_ ≈ k_L_ ≈ k, then the mismatch becomes Δα_actual_ = k_L_ × α_L,CLT_ − k_U_ × α_U,CLT_ ≈ k × (α_L,CLT_ − α_U,CLT_) ≈ k × Δα_CLT_. For the FEA displacement governed by Δα, the CLT-based prediction retains the correct sign and a reduced magnitude error, which is unlike the symmetric case, where the displacement is directly proportional to the absolute CTE and inherits the full multiplicative error.

## 4. Discussion

The asymmetric laminate architecture achieves two objectives [31,32,33] that symmetric designs cannot: it suppresses curvature-driven normal displacement by activating coupling stiffness [B] to generate a counterbalancing thermal moment, and it renders the displacement prediction robust to CLT inaccuracies through common-mode rejection. The 4.8-fold FEA underestimation of the symmetric baseline (43 µm predicted vs. 210 µm measured) originates from a systematic 1.75~2.38 × CTE underestimation by CLT. This error propagates linearly into displacement because symmetric designs are directly proportional to absolute CTE. This proportionality means that no single CTE calibration factor can correct the prediction across different laminate thicknesses, since the measured-to-predicted CTE ratio itself varies with ply count (2.38 for seven plies vs. 1.75 for nine plies).

By shifting the governing parameter from the mean CTE (α_mean_ = 1.628 × 10^−6^ K^−1^) to the same-batch CTE mismatch (Δα = 6.30 × 10^−7^ K^−1^), the asymmetric architecture fundamentally alters the deformation physics. The bimaterial beam mechanism produces a thermal moment proportional to Δα that opposes curvature-driven displacement, reducing thermal deformation by 50.4% (210 to 104 μm at +60 °C) and improving FEA accuracy from 79.4% to 4.8% error. The key robustness feature is common-mode rejection; since both skins share the same prepreg batch and cure cycle, the CLT prediction error (measured-to-CLT ratio 1.75–2.38) affects both skins similarly and is largely canceled in the mismatch term Δα = α_L_ − α_U_. Consequently, the displacement prediction for the asymmetric design retains the correct sign with a significantly reduced magnitude error, whereas the symmetric design inherits the full CLT systematic error. This is confirmed by Scheme 3’s validation results: the FEA prediction (109 μm) produced only 4.8% error relative to the experimental mean (104 μm) compared with 79.4% error for the symmetric baseline. The remaining discrepancy is attributable to the slightly different CLT error factors (k_U_ ≠ k_L_) arising from the thickness-dependent CTE variation (measured-to-CLT ratio: 2.38 for seven plies vs. 1.75 for nine plies), which prevents perfect common-mode cancelation.

It is appropriate to compare the proposed approach with existing methods while acknowledging their respective scopes. Variable-angle tow placement [12] optimizes the fiber path to achieve tailored thermal responses but requires specialized manufacturing equipment. Near-zero CTE laminates [13,14,15,16] use material hybridization to minimize the absolute expansion but may compromise other mechanical properties. Process-induced deformation compensation [17,18,19] adjusts the tooling geometry to counteract cure-induced distortions but does not address in-service thermal deformation. Active thermal control [20,21] maintains dimensional stability through heating elements but incurs power and mass penalties. These methods address different aspects of thermal deformation control, and not all are primarily intended to overcome uncertainties in CLT-based CTE prediction. The present approach is complementary: it exploits the manufacturing commonality between co-cured skins to make the displacement prediction robust to CTE uncertainty. However, the practical demonstration in this study is limited to a single rib geometry, and the quantitative effectiveness of the compensation will depend on the structural design (e.g., section height, skin thickness ratio). Extension to other curved CFRP structures with different geometries is a potential direction for future investigation rather than a conclusion established by the present data.

## 5. Conclusions

An asymmetric laminate architecture with deliberately retained coupling stiffness [B] outperforms the conventional symmetric design in controlling thermal deformation of arc-shaped CFRP antenna ribs. The asymmetric layup (9-ply upper, 7-ply lower and webs) creates a CTE mismatch of Δα = 6.30 × 10^−7^ K^−1^ between same-batch skins, activating [B] to generate a thermal moment that opposes curvature-driven normal displacement. A bimaterial beam model shows that the two skins share the same CTE error factor k, which factors out of the displacement expression through common-mode rejection, making the prediction robust to CLT inaccuracies. In a two-phase experiment (screening n = 3 per configuration; validation n = 9, M40J/BA9913, +60 °C), the asymmetric design achieved a dual objective: thermal deformation was reduced by 50.4% (210 → 104 µm) and FEA prediction accuracy improved from 79.4% to 4.8% error. This was accomplished using only conventional 0/90° prepreg and standard autoclave processing.

The present validation is limited to a single material system (M40J/BA9913), a single rib geometry (2100 mm chord, 1500 mm arc radius), and uniform +60 °C loading. Future work [34,35,36] should extend the approach in three directions: (a) continuous optimization of the ply-count asymmetry through surrogate modeling to identify the optimal configuration; (b) validation under spatially non-uniform thermal boundary conditions representative of low Earth orbit [37]; and (c) assessment of long-term cyclic thermal stability and space-environment degradation (UV radiation, atomic oxygen) on CTE and laminate integrity [38]. The principle of common-mode rejection via same-batch CTE mismatch may extend to other curved CFRP structures where symmetric designs offer no built-in compensation mechanism, subject to structural-specific validation.

## Figures and Tables

**Table 1:** M40J/BA9913 UD-ply properties.

Property	Symbol	Value
Longitudinal modulus	$E_{1}$	227,600 MPa
Transverse modulus	$E_{2}$	5800 MPa
Shear modulus	$G_{12}$	2850 MPa
Poisson’s ratio	$\nu_{12}$	0.35
Longitudinal CTE	$\alpha_{1}$	−4.95 × 10^−7^ K^−1^
Transverse CTE	$\alpha_{2}$	3.15 × 10^−5^ K^−1^
Ply thickness	t	0.11 mm

**Table 2:** Measured vs. CLT-predicted laminate CTE.

Layup	CLT/E_1_ (MPa)	CLT/E_2_ (MPa)	Measured/α_1_ (K^−1^)	CLT/α_1_ (K^−1^)	Ratio
7-ply	1.01 × 10^5^	1.33 × 10^5^	1.943 × 10^−6^	8.15 × 10^−7^	2.38
8-ply	1.17 × 10^5^	1.17 × 10^5^	1.052 × 10^−6^	5.60 × 10^−7^	1.88
9-ply	1.05 × 10^5^	1.29 × 10^5^	1.313 × 10^−6^	7.52 × 10^−7^	1.75

**Table 3:** FEA-predicted displacement at +60 °C and comparison with screening experiments.

Scheme	Layup (Upper/Lower/Web)	FEA (μm)	Exp. Mean (μm)	∆ (μm)	Rel. Dev. (%)
Baseline	7/7/7	43.3	209.7	166.4	79.4
Scheme 2	8/7/7	324.0	167.1	157.0	93.9
Scheme 3	9/7/7	109.0	131.0	22.0	16.8

**Table 4:** Per-specimen displacement for Scheme 3 at +60 °C.

Specimen	D01	D02	D03	D04	D05	D06	D07	D08	D09
δ (μm)	74	99	114	89	61	112	165	114	109

**Table 5:** FEA vs. experiment values for Scheme 3.

Metric	FEA (μm)	Exp. Mean (μm)	Abs. Dev. (μm)	Rel. Dev. (%)
δ	109	104	5	4.8

## Data Availability

The original contributions presented in this study are included in the article. Further inquiries can be directed to the corresponding author.
